# Hierarchical Deep Learning for Abnormality Classification in Mouse Skeleton Using Multiview X-Ray Images: Convolutional Autoencoders Versus ConvNeXt

**DOI:** 10.3390/jimaging11100348

**Published:** 2025-10-07

**Authors:** Muhammad M. Jawaid, Rasneer S. Bains, Sara Wells, James M. Brown

**Affiliations:** 1 School of Engineering & Physical Sciences, College of Health & Science, University of Lincoln, Brayford Pool, Lincoln LN6 7TS, UK; jamesbrown@lincoln.ac.uk; 2 Mary Lyon Centre at MRC Harwell, Oxfordshire OX11 0RD, UK

**Keywords:** mouse phenotyping, skeletal abnormality, hierarchical learning, multiview representation, convolutional autoencoder

## Abstract

Single-view-based anomaly detection approaches present challenges due to the lack of context, particularly for multi-label problems. In this work, we demonstrate the efficacy of using multiview image data for improved classification using a hierarchical learning approach. Using 170,958 images from the International Mouse Phenotyping Consortium (IMPC) repository, a specimen-wise multiview dataset comprising 54,046 specimens was curated. Next, two hierarchical classification frameworks were developed by customizing ConvNeXT and a convolutional autoencoder (CAE) as CNN backbones, respectively. The customized architectures were trained at three hierarchy levels with increasing anatomical granularity, enabling specialized layers to learn progressively more detailed features. At the top level (L1), multiview (MV) classification performed about the same as single views, with a high mean AUC of 0.95. However, using MV images in the hierarchical model greatly improved classification at levels 2 and 3. The model showed consistently higher average AUC scores with MV compared to single views such as dorsoventral or lateral. For example, at Level 2 (L2), the model divided abnormal cases into three subclasses, achieving AUCs of 0.65 for DV, 0.76 for LV, and 0.87 for MV. Then, at Level 3 (L3), it further divided these into ten specific abnormalities, with AUCs of 0.54 for DV, 0.59 for LV, and 0.82 for MV. A similar performance was achieved by the CAE-driven architecture, with mean AUCs of 0.87, 0.88, and 0.89 at Level 2 (L2) and 0.74, 0.78, and 0.81 at Level 3 (L3), respectively, for DV, LV, and MV views. The overall results demonstrate the advantage of multiview image data coupled with hierarchical learning for skeletal abnormality detection in a multi-label context.

## 1. Introduction

Musculoskeletal disorders (MSDs) are the second most common cause of disability worldwide and can affect different parts of the body. The recent global statistics show that around 1.7 billion people suffer from MSDs, which often lead to severe disability and long-term pain [1]. Interestingly, these disorders cover a wide range of conditions involving the issues related to bones, joints, muscles, ligaments, and tendons. From a clinical perspective, an accurate and efficient diagnosis of MSDs is essential for pain management and effective treatment. It is important to mention that along with aging and injury, genetic and hereditary factors play a significant role in the development of MSDs, frequently resulting in skeletal abnormalities. Accordingly, understanding these underlying genetic risks is a major focus of current research. Since direct investigation in humans can be difficult, researchers frequently turn to animal models as described in [2]. Among these, mouse models are especially valuable because they reproduce quickly, share important similarities in musculoskeletal structure, and have a high degree of genetic resemblance to humans.

A number of different imaging modalities are being used for the visualization of internal dynamics. Specifically, in the context of structural and skeletal abnormalities, the frequently used imaging modalities include X-rays and computed tomography (CT) imaging. Plain radiography (X-ray) plays a significant role in the diagnosis of musculoskeletal disorders by providing clear visualization of skeletal structures. The images require less time and provide detailed insights into the skeleton, helping clinicians to identify fractures, dislocations, deformities, and other pathological changes, thereby supporting accurate diagnosis and guiding interventional procedures. With the availability of specialized equipment and imaging protocols, researchers and veterinary practitioners have reported the use of X-ray imaging to study musculoskeletal health in laboratory mouse models [3], which are commonly used for phenotyping and for gaining insights into human disease. Building on this potential, the research community has increasingly focused on developing automated systems for the detection of skeletal abnormalities in the acquired X-ray images. However, the progress in this area has been limited, largely because two-dimensional plain X-rays provide restricted information, and symptoms often overlap in single-view images. Therefore, we propose an automated system that uses multiview representations (integration of multiview X-ray images for compact representation of specimens), enabling the extraction of richer and more discriminative features to address the complex and multi-label nature of skeletal abnormality detection.

After the brief introduction, we now present the organization of this paper. Beginning with the introduction, we present an overview of related works in Section 2. This is followed by dataset curation details since IMPC data originates from multiple sources and inherently comes with various inconsistencies. We then explain the procedure for building a compact specimen-wise multiview representation. In the subsequent section, we present implementation details for multiview hierarchical classification using two architectures, i.e., ConvNeXt and a convolutional autoencoder. Finally, the performance of the two architectures is presented, where we demonstrate the efficacy of multiview data-based classification in comparison with any single view. Moreover, the performance of the architecture is validated using class activation maps for the interpretation of model decisions.

## 2. Related Work

With the emergence of high-resolution, digitized medical imaging and technical computing advancements, automated diagnosis systems are emerging as the tool for modern healthcare. In this context, numerous studies have highlighted the potential of deep learning to enhance clinicians’ ability to interpret X-ray images. The following section presents a brief review of studies focusing on automated X-ray interpretation, skeletal abnormality detection, and hierarchical learning.

A recent work by [4] has reported the use of weakly supervised learning for the detection of skeletal abnormalities from musculoskeletal X-rays. The authors employed a sequential approach, i.e, potential regions were localized first, and subsequently abnormality detection models were trained using the identified regions. Although the authors reported a detection accuracy of 97.9%, the main limitation was the use of localized human X-ray images from the MURA (Musculoskeletal Radiographs) dataset, which significantly differs from full-body mouse X-rays. Another application of deep learning for the detection of bone fractures was reported by [5]. A promising detection accuracy of 92.44% was reported for the proposed model based on X-ray images; however, the scope of the problem was only binary, i.e., the aim was to differentiate fractured bone from healthy bone. Furthermore, the image dataset consisted of only 100 images, which limits the usability of the approach and does not exploit the true potential of deep neural networks. Similarly, a number of existing deep learning architectures, including Xception, Inceptionv3, VGG-19, DenseNet, and MobileNet were evaluated by [1] for automatic interpretation of X-ray images from the MURA dataset. The results indicated competitive performance of the existing models for binary classification; however, multiple anomalies were not detected, suggesting the proposed models have highly limited practicality.

Few published works have specifically addressed the use of deep learning for the interpretation of mouse X-rays. An interesting work reported by Babalola et al. [6,7] employed convolutional neural networks (CNNs) for binary classification of specimen gender. With a conventional VGG16 backbone, the proposed model was trained on a total of 10,400 X-ray images for sex classification. According to the authors, a promising accuracy of 98% was achieved in distinguishing between male and female mice. Moreover, class activation maps (CAM) [8] were presented to conclude that the chest and the pelvic regions were utilized by the CNN model to label specimens on the basis of sex. Although this work is among the first to use a CNN for mouse X-ray interpretation, the scope of this work was again limited to binary labeling. To summarize, automated detection of bone abnormalities has been widely attempted, and many works have been reported in the literature [9,10,11]; however, the reported works operated on either limited image data or were confined to binary labeling. Moreover, a comprehensive evaluation of reported works has been presented in [12] with a focus on particular bones being investigated and the proposed deep learning architecture.

Likewise, several interesting works [13,14,15] have utilized X-ray images for the automated abnormality detection; however, the main focus of these works was the detection of pathological abnormalities. These studies demonstrated promising performance, with mean AUC scores of 0.810, 0.764, and 0.840, respectively. However, they relied on single-view chest X-rays from the CheXpert [16] dataset, which provides limited information. Another interesting work in this context was reported by Liu et al. [17], who employed an incremental learning strategy, i.e., training the model gradually from common to rare abnormalities as per clinical practice. According to the results, the model achieved a promising mean AUROC of 0.889 on the CheXpert dataset; however, single-view data and customized selection functions were employed, including correlation and similarity. The use of transfer learning for X-ray-based abnormality detection was reported in [18], i.e., the authors employed 16 CNN architectures (pre-trained on ImageNet) for the interpretation of chest X-rays. The results demonstrated that while a transfer learning approach is used, truncating layers can lead to enhanced performance in terms of improved parameter efficiency and the model’s accuracy. Likewise, Huang et al. [19] also experimented with X-ray-based multi-label classification using CNN backbones. The results demonstrated that ImageNet-based weights led to improved training of the model based upon learned features. Moreover, it was also demonstrated that the integration of image data from multiple sources led to improved model performance. Wehrmann et al. [20] examined multi-label problems from a structural perspective, using hierarchical multi-label classification networks (HMCNs). In comparison to conventional direct learning, HMCN exploits hierarchical relations in the learning process. Although promising results were reported by the authors, the model’s suitability was not demonstrated for medical imagery. Similarly, Asadi et al. [21] recently explored the potential of hierarchical classification; however, their study detected clinical abnormalities when only single-view chest X-ray images were used.

In brief, the existing research highlights the potential of deep learning for musculoskeletal and X-ray–based abnormality detection, yet there exist several limitations. While weakly supervised and CNN-based approaches have yielded high accuracies on human datasets such as MURA and CheXpert, most studies are restricted to binary classification tasks, rely on limited or single-view data, and often focus on specific bones or pathologies. Interestingly, applications to mouse X-rays are even more limited, with work to date largely confined to tasks such as gender classification rather than abnormality detection. This indicates a clear gap and the need for more comprehensive approaches capable of handling multi-view, multi-label, and complex abnormality detection using X-ray images. In this context, we believe that a hierarchical approach is highly suitable for medical image analysis, i.e., abnormality detection level by level as per clinical practice. A small-scale study in this context was conducted by the authors [22], where the potential of hierarchical learning was demonstrated using single-view X-ray image data. Hence, in this study, we investigated the potential of multiview representation of mouse specimens to enhance skeletal abnormality detection performance. To the best of our knowledge, this is the first report of such an approach.

## 3. Materials and Methods

Starting with the data collection, preprocessing and initial analysis were performed to label the data at different hierarchical levels as explained in Section 3.1 and Section 3.2. The initial analysis revealed a number of inconsistencies across participating centers, including intensity/brightness variations, field of view, and number of images per specimen. Moreover, it was revealed that certain views were less prevalent in comparison with others. Subsequently, the specimen-wise multiview dataset was curated as explained in Section 3.3. The following section explains the hierarchical models built on top of two backbones for musculoskeletal abnormality detection, i.e., ConvNeXt and a convolutional autoencoder (CAE). Using a multiview representation of each specimen, the hierarchical approach segregates *abnormal* specimens at the top level. It then differentiates between three subclasses of *abnormal* at the second level, further refining them into 10 specific categories at the third level. The efficacy of the multiview image data for improved classification of abnormalities is demonstrated in Section 5, with a higher mean AUC for multiview data over any single view.

### 3.1. Data Collection and Initial Analysis

**Figure 1:**
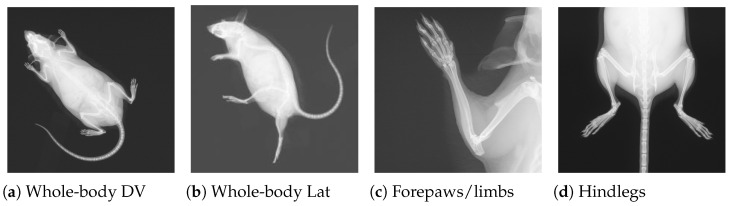
Four X-ray images for a single mouse from the IMPC image dataset. Views (**a**,**b**) are acquired for all specimens, whereas (**c**,**d**) are only acquired if necessary.

The image data (54,830 specimens and 261,605 images [2]) for this research were obtained from the public repository of the International Mouse Phenotyping Consortium (IMPC) [2]. The International Mouse Phenotyping Consortium (IMPC) is an ongoing multi-institutional project aimed at producing a catalog of mammalian gene function through standardized screens of single-gene-knockout mice [23]. It is worth noting that manual phenotyping of skeletal abnormalities is extremely laborious (i.e., up to 53 observations per specimen), and it becomes much harder if a gene leads to multiple skeletal anomalies. The X-ray imaging is one of several procedures carried out at IMPC as part of a high-throughput phenotyping pipeline, as explained in [24]. To investigate the impact of gene alteration on the whole skeletal system, images are often acquired from different views/perspectives, including dorsoventral (DV), lateral (LV), skull, forelimbs, and hindlimbs, as demonstrated in Figure 1.

The view-wise distribution of the obtained image data is illustrated in Table 1, where it can be observed that full-body views (DV and LV) have a higher number of images, followed by skull images. Based on the fact that the majority of the skeletal information is captured in the full-body view, all specimens were imaged from two viewpoints, whereas the remaining views were only captured as required. For instance, if a suspicion was detected in the full-body view, then additional images of the skull were acquired to mark potential anomalies in the skull. Similarly, suspected specimens were also imaged for forelimbs and hindlimbs to rule out associated anomalies. In addition to the total number of images for a particular view, an interesting factor is the subsequent split into two classes, i.e., normal versus abnormal. It can be observed from the *fourth* column of the table that two full-body views represent adequate number of abnormal images, whereas the remaining four views contain very few abnormal cases.

**Figure 2:**
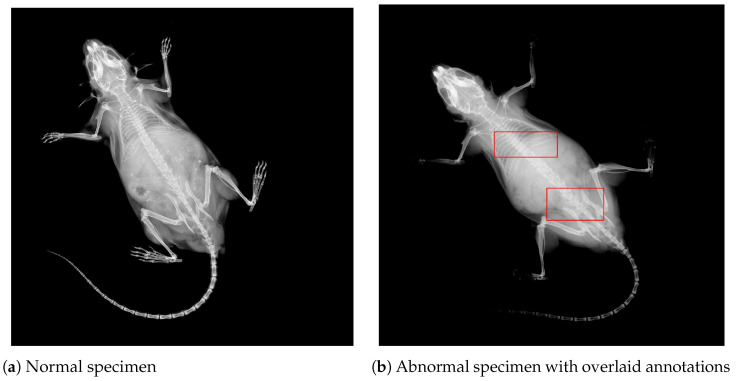
DV images from the IMPC dataset for two different specimens. Image contrast has been adjusted to highlight areas with potential abnormalities. (**a**) A normal mouse. (**b**) An abnormal mouse with overlaid bounding boxes, showing vertebral fusion and caudal spine abnormalities, respectively.

Moreover, it is worth noting that the 1412 abnormal DV images contain 3127 marked structural abnormalities. This leads to the conclusion that images contain multiple skeletal anomalies simultaneously, which is expected given the systemic effects of single-gene knockouts, i.e., gene suppression lead to multiple structural abnormalities at different locations. For visual comprehension, contrast-enhanced images for two specimens are presented in Figure 2. The left panel in Figure 2a shows a DV view labeled as normal, whereas the right panel presents an abnormal specimen. It can be observed from Figure 2b that there exist multiple abnormalities, including vertebral fusion and caudal deformation.

### 3.2. Preprocessing and Specimen-Wise Labeling

**Figure 3:**
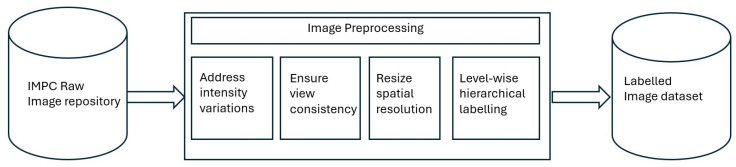
Flowchart demonstrating the preprocessing activities performed for dataset curation. The preprocessing included addressing intensity variations and spatial resolution differences.

Once the image data was gathered, preprocessing and hierarchical labeling were performed as explained in Figure 3. Since the image data originated from different phenotyping centers, there existed differences in terms of image quality (brightness, intensity, and spatial resolution). Accordingly, image data was processed to curate an adequate dataset for the multi-label classification task. In the first step, filtration was performed to ensure consistency and quality of the dataset. Specifically, we removed images with sharp intensity variations, different acquisition angles, or limited fields of view (FoVs). All images were then resized to 224×224×3.

In the context of three-level hierarchical labeling of individual specimens, it is important to mention that IMPC labels up to 53 individual anomalies as defined in [23]. This manual phenotyping process takes into account all possible deformations in body structure from the skull to the distal caudal region. However, it becomes very challenging to employ all 53 labels and subsequently detect associated abnormalities due to the correlation of abnormalities, i.e., multiple abnormalities are frequently present in one image. Hence, a structured labeling approach was applied in this study. At the top level, a particular specimen was categorized into *normal* or *abnormal* depending upon the overall screening; i.e., L1_label was set as *abnormal* if there existed any one of the 53 expert-derived abnormalities and *normal* otherwise, as expressed in Equation (1).

Let

- $I=\{i_{1},i_{2},\ldots ,i_{n}\}$ be the set of input images.
- $A=\{a_{1},a_{2},\ldots ,a_{53}\}$ be the set of expert-annotated abnormalities.
- $f(i_{k})\subseteq A$ denote the set of abnormalities present in image $i_{k}$.

(1) $$L1\_label\left(i_{k}\right)=\left\{\begin{array}{cc} \mathrm{abnormal}, & \mathrm{if}|f(i_{k})|\geq 1\\ \mathrm{normal}, & \mathrm{otherwise} \end{array}\right.$$

For the L2_label, 53 standard abnormalities were combined into subclasses based upon locality and nature of abnormality, leading to five possibilities, i.e., *Limbs, Ribcage, Skull, Spine, and Whole-body*. The *Limbs* subclass covers abnormalities related to digit integrity, *femur, fibula, humerus, tibia, ulna, or syndactylism*. The subclass *Ribcage* reflects abnormalities regarding *rib fusion, number of ribs, shape of ribs, and rib morphology*. The *skull* subclass addresses abnormalities including *mandibles, maxilla/pre-maxilla skull, teeth, and zygomatic bone*, whereas subclass *Whole-body* represents abnormalities affecting the *clavicle, scapulae, pelvis, and joints*. Finally, the most important and highly prevalent subclass *Spine* covers the majority of structural abnormalities, including *kyphosis, lordosis, scoliosis, spine shape, morphology, and vertebral anomalies*. The mathematical representation for L2_label is defined in Equation (2)

(2) $$L2\_label\left(i_{k}\right)=\left\{g_{2}\left(a\right)|a\in f\left(i_{k}\right)\right\}$$

where $g_{2}:A\to C_{2}$ is a mapping from each abnormality to its level_2 subclass, with $C_{2}=\left\{\mathrm{Limbs},\mathrm{Ribcage},\mathrm{Skull},\mathrm{Spine},\mathrm{Whole}\text{-}\mathrm{body}\right\}$ containing subclasses.

**Figure 4:**
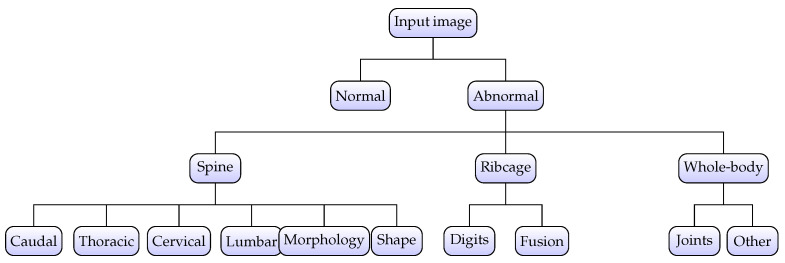
Hierarchical labeling for specimens. The abnormality is identified at the top level and subsequently classified into subclasses at level_2 and level_3, respectively.

In contrast with L2_label, the third level of the hierarchical model performs granular classification for more localized identification as demonstrated by Equation (3). Accordingly, Level 2 abnormalities were further divided into subcategories to reflect the precise and targeted location of the abnormality.

(3) $$L3\_label\left(i_{k}\right)=\left\{g_{3}\left(a\right)|a\in f\left(i_{k}\right)\right\}$$

where $g_{3}:A\to C_{3}$ is the mapping from each abnormality to its level_3 subclass, with $C_{3}=\left\{\mathrm{Caudal},\mathrm{Thoracic},\mathrm{Cervical},\mathrm{Lumbar},\mathrm{Morphology},\mathrm{Shape},\mathrm{Digits},\mathrm{Fusion},\mathrm{Joints},\mathrm{Other}\right\}$ containing subclasses. The hierarchical labeling process is visually presented in Figure 4 where it can be observed that level_1 identifies abnormal samples whereas level_2 and level_3 further categorize abnormality into specific subclasses.

### 3.3. Multi-View Dataset Curation

After image-wise labeling, the subsequent step was to develop a multiview dataset, i.e., a representation of individual specimens using X-rays from different views. It can be observed from Table 1 that two full-body views (DV and LV) comprise a significant number of images. In contrast, the remaining views contain comparatively fewer images as they focus on a limited region of the body, i.e., skull, forelimbs, or hindlimbs. Moreover, it can also be observed from the table that almost all structural abnormalities were reflected in full body view, with rare occurrences in the remaining four views. Therefore, the two full-body views were chosen to represent a specimen in the context of multiview analysis, while the remaining four views were discarded. Accordingly, a total of 166,897 images containing 8237 skeletal abnormalities were selected for full-body representation of individual specimens. The selected images from two views were synchronized using *Biological_sampleID* to correlate views for a particular specimen, leading to 54,046 unique specimens (52,340 normal versus 1706 abnormal).

**Figure 5:**
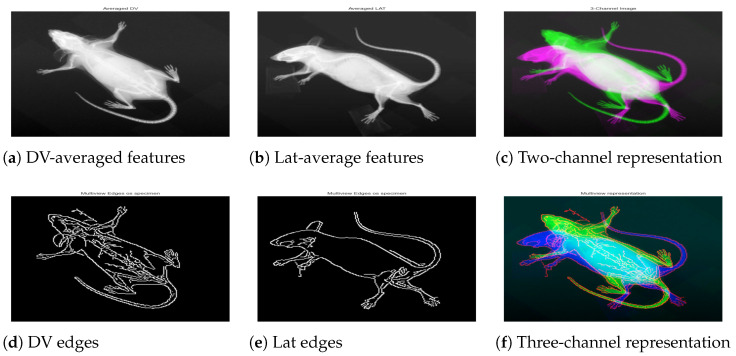
A specimen with biological sample ID 183808. The first row shows mean representation for DV (**a**), Lat (**b**), and the two-channel image (**c**). The second row shows the individual edge map for respective views (**d**,**e**), and the three-channel image (**f**) constructed by integrating the cleaned edge map into the two-channel image.

The specimen-wise compact representation was generated by combining DV, LAT, and the respective edge-map. For a particular *Biological_sampleID*, where there existed multiple images from the same viewpoint, the “mean” was computed to produce a single image per view as demonstrated in Figure 5a,b. The resultant mean helped reduce noise and highlighted common features across multiple instances, leading to a two-channel image of Figure 5c. Next, edge features were obtained using the Canny algorithm for two views, and an integrated edge map was constructed as demonstrated in Figure 5d,e. To combine the edges from two views meaningfully, we applied weighted integration, which blended the edge maps from the LAT and DV images with slightly higher weights for DV. This allowed the blending of the edges into an integrated map while giving more importance to DV due to the easy structural interpretation in DV. The result was an integrated edge map that captured the most prominent features from both LAT and DV images. Finally, a three-channel multiview compact representation for the specimen was obtained by stacking the mean images with the edge map, as demonstrated in Figure 5f.

The multi-view dataset curation was followed by specimen-wise labeling, such that every specimen was assigned hierarchical labels based on the union of those assigned at the image level. Due to the limited occurrence of abnormalities in the skull and limbs subclasses, these categories were excluded from the analysis. Consequently, the final dataset consisted of 1651 abnormal specimens, with the subclass distribution presented in Table 2. This refinement resulted in three subclasses at level_2 and ten subclasses at level_3, as illustrated in Figure 4. Accordingly, the adopted labeling approach yielded a 1 × 13 vectorized representation for each specimen; i.e., a *normal* specimen had all *zeros*, whereas an *abnormal* specimen had at least one non-zero entry corresponding to abnormalities at level_2 and level_3.

## 4. Multi-View Hierarchical Classification

**Figure 6:**
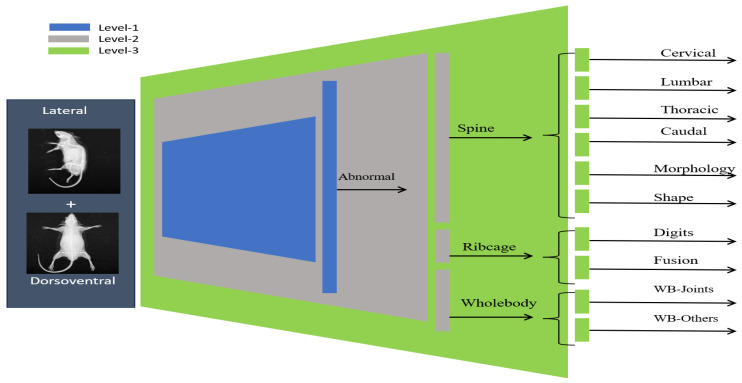
Overview of the hierarchical classification framework. The three-level model performs classification with progressively increasing anatomical granularity.

The overall concept of the multiview hierarchical implementation is presented in Figure 6; i.e., multiview image data is provided as an input, and the hierarchical models output a 1 × 13 vector representing specific abnormalities. The hierarchical model was built on top of two state-of-the-art backbone architectures: ConvNeXt [25] and convolutional autoencoder (CAE) [26] as described in this section.

### 4.1. Multiview Hierarchical Classification Using ConvNeXT

**Figure 7:**
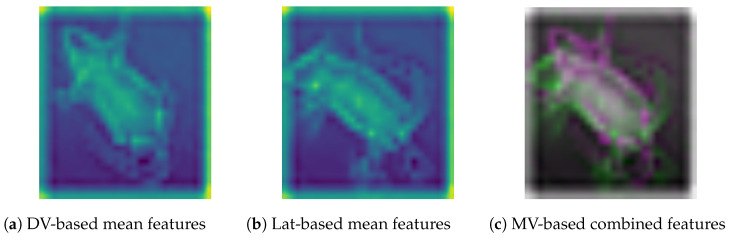
Visual representation of EfficientNet-based extracted features from DV, Lat, and the subsequent combined feature map for hierarchical classification.

In the first step, we employed the ConvNeXt backbone for hierarchical classification; however, the base model was modified to fully exploit the multi-view image data. Since the input image contains information for multiple views, the extraction of descriptive features for effective model training presents a challenge. Accordingly, we investigated different feature extraction techniques to integrate multiview information, including conventional averaging, min-max projection, principal component analysis (PCA), and gradient representation; however, empirical experimentation led to subpar results, emphasizing the need for sophisticated feature extraction techniques. Subsequently, we employed a convolutional approach, namely EfficientNetV0 [27], for effective feature extraction. This CNN-based feature extraction technique is often applied for extracting and aggregating high-level features from images, which are highly useful for classification and visualization. Accordingly, dorsoventral and lateral images were processed using EfficientNet, meaningful features were extracted from *block3b-dwconv* layer, and a subsequent combined features map was obtained, as shown in Figure 7.

After a multiview feature map was generated, the representative feature map of Figure 7c was input to the first level of a hierarchical classification model. Level_ 1 model takes an integrated feature map as an input and subsequently performs binary classification to segregate *abnormal* specimens from *normal*. The hierarchical architecture was built by initializing the ConvNeXtBase model, which was pre-trained using the ImageNet dataset. The input shape was set as 224 × 224, and the top layer of the model was removed for customization. The extracted features from the base model (second last layer of ConNeXTBase) with shape (7 × 7 × 1024) were passed through a global average pooling layer for dimensionality reduction, followed by a dense layer with 32 neurons and ReLU activation. The last (output) layer employed a single neuron with a sigmoid activation function to segregate between *normal* and *abnormal* specimens. The model was compiled with the *Adam* optimizer [28], using a custom learning rate of 0.001, and the *binary cross-entropy* loss function. In addition, we applied validation loss-based early stopping phenomena to avoid overfitting, and the model was trained for 100 epochs using a train–validation split for optimal performance.

The level_1 model was extended with an additional block to classify *abnormal* specimens into three subclasses, i.e., *Spine*, *Ribs*, and *Whole-body*. Accordingly, the output from the second-last layer of model_level1 was directly passed as an input to block2, where the vector was reshaped, and subsequently, convolution and global averaging operations were applied. The block2 convolutional layer (”Conv2D” with ReLU activation) was used for refined feature learning, whereas max-pooling was employed for dimensional reduction. The resultant feature map was then passed through three fully connected layers (”Dense” with ReLU activation and L2 regularization) to exploit internal relationships. The final layer employed three neurons with a sigmoid activation function to enable multi-label classification by making independent predictions for three subclasses. Moreover, the *Adam* optimizer was used with a custom learning rate of ”0.0001”, and the model was compiled with binary cross-entropy loss. Accordingly, the level_2 model employs the output of model level_1, i.e., already learned features, and adjusts weights to classify *abnormal* specimens into three subclasses.

**Figure 8:**
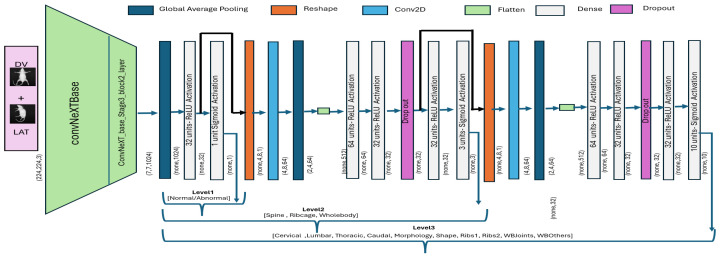
ConvNeXt-driven hierarchical classification model. The output of one block is provided as input to the subsequent block for subsequent classification.

The output from the second-to-last layer of level_2 was reshaped into a 3D tensor and subsequently passed as an input to model level_3. The convolutional layers for block3 were then applied to capture refined spatial features. The convolutional layer (Conv2D with ReLU activation) was followed by a max-pooling and flattening layer. Subsequently, three fully connected layers with ReLU activation and L2 regularization were added to exploit internal relationships. The final layer employed 10 neurons with a sigmoid activation function. The model was compiled using Adam optimizer with a custom learning rate (0.00011), and a binary cross-entropy loss function. As the level_2 model was already trained over the mice dataset, the layers were frozen to retain the learned feature representations and prevent overfitting. The overall architecture for the hierarchical classification model is presented in Figure 8, whereas the mathematical formulation is defined in Equations (4)–(6).

(4) $$y_{\mathrm{level}1}=\sigma \left(W_{2}\cdot \mathrm{ReLU}\left(W_{1}\cdot \mathrm{GAP}\left(f_{\mathrm{base}}\left(x\right)\right)+b_{1}\right)+b_{2}\right)$$

where the input $x\in R^{224\times 224\times 3}$ represents the image input, $f_{\mathrm{base}}$ defines the ConvNeXt-based features, and (GAP) reflects the global average pooling operation. Moreover, $W_{1}$ and $W_{2}$ represent weights for two dense layers with respective bias terms of $b_{1}$ and $b_{2}$. The output layer employs sigmoid activation σ(·) for binary classification.

(5) $$\begin{array}{cc} y_{\mathrm{level}2}=\sigma ( & W_{6}\cdot \mathrm{ReLU}(W_{5}\cdot \mathrm{ReLU}(W_{4}\cdot \mathrm{ReLU}(W_{3}\cdot \mathrm{Flatten}(\mathrm{MaxPool}(\mathrm{Conv}2D\\ \; & \left(\mathrm{Reshape}\left(h_{1}\right)\right)))+b_{3})+b_{4})+b_{5})+b_{6}) \end{array}$$

where the input feature vector $h_{1}$ is derived from the level_1 model, Reshape(·) defines a reshape operation, and Conv2D represents a convolution layer. Moreover, MaxPool and Flatten represent custom operations and $W_{3}$, $W_{4}$, $W_{5}$, and $W_{6}$, represent weight matrices for dense layers. The output layer employs sigmoid activation as $y_{\mathrm{level}2}$, which is a multi-label classification.

(6) $$\begin{array}{c} y_{\mathrm{level}3}=\sigma (W_{10}\cdot \mathrm{ReLU}(W_{9}\cdot \mathrm{ReLU}(W_{8}\cdot \mathrm{ReLU}(W_{7}\cdot \mathrm{Flatten}(\mathrm{MaxPool}(\mathrm{Conv}2D\\ \left(\mathrm{Reshape}\left(z_{2}\right)\right)))+b_{7})+b_{8})+b_{9})+b_{10} \end{array}$$

where $z_{2}$ is the output from the model_level2, Reshape(·) defines reshape operation, and Conv2D represents the convolutional layer. Moreover, MaxPool and Flatten represent custom operations, and $W_{7}$, $W_{8}$, $W_{9}$, and $W_{10}$ represent weight matrices for dense layers. The output layer employs sigmoid activation as $y_{\mathrm{level}3}$, which is a multi-label classification.

### 4.2. Multiview Hierarchical Classification Using CAE

In addition to the ConvNeXt-based hierarchical architecture, we also implemented a variant of a convolutional autoencoder to demonstrate the advantage of the multi-view data. The standard CAE architecture comprises two parts; i.e., the encoder compresses the input data to a lower-dimensional latent space (encoding) by extracting meaningful features, whereas the decoder reconstructs the input data from the compressed representation. Accordingly, this process forces the network to learn a meaningful representation of the data in the latent space. Although autoencoder is an unsupervised approach primarily used for dimensionality reduction and data reconstruction, we employed this technique for efficient feature extraction in the context of hierarchical classification.

**Figure 9:**
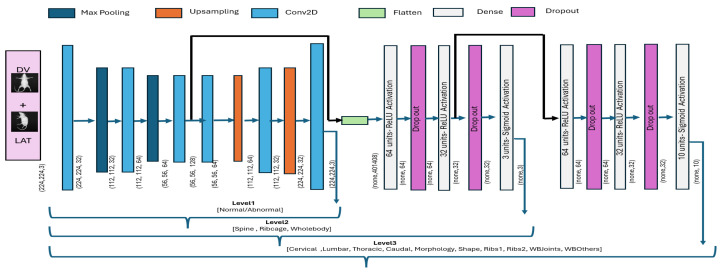
Autoencoder-based hierarchical classification. The output of one block is provided as input to the subsequent block for hierarchical classification.

Accordingly, we used the autoencoder image reconstruction part for feature extraction, i.e., level_1 classification, and a subsequent hierarchical model was built using learned features, as demonstrated in Figure 9. Starting with a 2D convolutional layer, a max-pooling operation was applied to progressively reduce the spatial dimensions of the input image. The final convolutional layer was defined with a spatial resolution of 56 × 56 and 128 filters. The decoder part reflected the reverse process, where the compressed features were upsampled to reconstruct the original image. The autoencoder model was trained end to end using the mean- squared-error (MSE) loss defined in Equation (7). Once the model was trained to converge, we evaluated model performance over the test subset using standard metrics of reconstruction error and structural similarity. The reconstruction performance is very crucial as it serves as level_1, and the subsequent hierarchical classification directly depends upon the feature extraction capability of the model.

(7) $$\mathrm{MSE}=\frac{1}{N}\sum_{i=1}^{N}{\left(X_{\mathrm{input}}\left(i\right)-X_{\mathrm{reconstructed}}\left(i\right)\right)}^{2}$$

where *N* denotes the total number of pixels in the input image. The term $X_{\mathrm{input}}\left(i\right)$ represents the *i*-th pixel of the original input image, and $X_{\mathrm{reconstructed}}\left(i\right)$ corresponds to the *i*-th pixel of the reconstructed image generated by the model.

**Figure 10:**
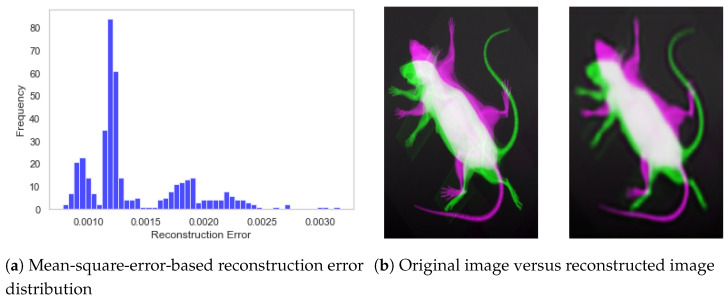
Performance of the convolutional autoencoder model. (**a**) Distribution of reconstruction error for the test subset. (**b**) Visual representation of original and reconstructed images.

The performance of the encoder model is presented in Figure 10, which shows the overall distribution of reconstruction error and a sample specimen from the dataset. It can be observed from Figure 10a that the reconstruction error remained stable within the range of 0.000 to 0.003, with a mean value of 0.00139 and a standard deviation equivalent to 0.0004. Similarly, the visual representation of Figure 10b also demonstrates the CAE’s capability to extract meaningful features and subsequently reconstruct the image.

**Figure 11:**
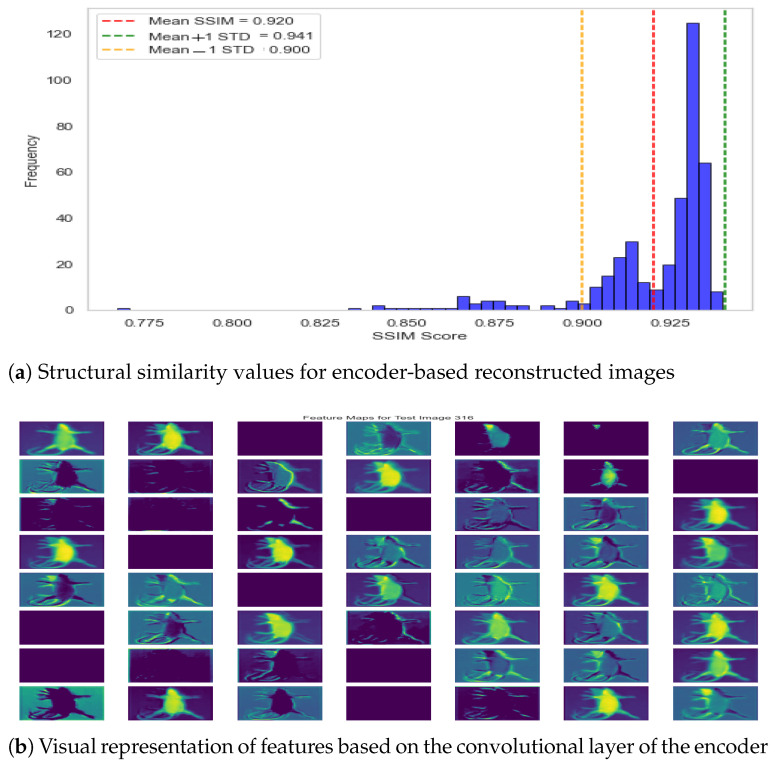
Autoencoder performance—reconstruction error and feature visualization.

In addition to visual comparison of the reconstructed image, we also computed the structural similarity score between two instances, i.e., actual versus reconstructed. The structural similarity score for the test dataset is presented in Figure 11a, where it can be observed that structural similarity remained greater than 90% for almost all specimens with a few exceptions in the range of 80%. The mean SSIM score of 0.9204 demonstrates the exceptional performance of the autoencoder model, and the standard deviation equivalent to 0.0203 shows consistent performance across the dataset. Moreover, it shows that the model has learned descriptive features from the image, and the subsequent feature map can be used for effective classification in the hierarchical model. The underlying dynamics of the autoencoder model are also presented in Figure 11b, which shows the feature map from the second-to-last layer. Accordingly, it can be observed that the model learned structural features by exploiting different regions of the specimen.

In the context of hierarchical classification, the feature map generated by encode (level_1) was used as an input to the level_2 model, as demonstrated in Figure 9. Since representative features were well identified by the encoder, we applied a flattening operation, subsequently followed by fully connected (Dense) layers. It is important to note that additional convolutional layers are typically not applied to encoder-based features, as convolution has already been performed during the encoding process. Accordingly, the level_2 architecture consisted of two hidden layers, with ReLU activation and dropout for regularization, followed by an output layer with three neurons and sigmoid activation for level_2 classification. The level_2 model was compiled using a standard Adam optimizer with a small learning rate (0.00001) and binary cross-entropy as the loss function, which is suitable for multi-label classification tasks. In addition, we employed early stopping based on validation loss. Similarly, the level_3 model was constructed by extending level_2, where an additional set of layers was appended to enable level_3 classification. These appended layers consisted of two fully connected layers with dropout to reduce overfitting, followed by an output layer containing multiple neurons with sigmoid activation to support multi-label classification. The model was optimized using binary cross-entropy loss function in the Adam optimizer, with early stopping used to avoid overfitting. The visual illustration for the CAE-based hierarchical model is presented in Figure 9, whereas the mathematical representation for the two hierarchical levels is defined by Equations (8) and (9). Moreover, the hyperparameter configuration for two architectures is demonstrated in Table 3 for a quick review.

(8) $$y_{\mathrm{level}2}=\sigma \left(W_{3}\cdot \mathrm{ReLU}\left(W_{2}\cdot \mathrm{ReLU}\left(W_{1}\cdot X_{\mathrm{encoded}}+b_{1}\right)+b_{2}\right)+b_{3}\right)$$

where the input $X_{\mathrm{encoded}}$ is the encoder-based features, and $W_{1}$,$W_{2}$, and $W_{3}$ represent weights for three dense layers with the respective bias terms of $b_{1}$, $b_{2}$, and $b_{3}$. Moreover, σ(·) represents the sigmoid activation function used in the output layer to address multi-label classification

(9) $$y_{\mathrm{level}3}=\sigma (W_{6}\cdot \mathrm{ReLU}(W_{5}\cdot \mathrm{ReLU}(W_{4}\cdot X_{\mathrm{level}2\_\mathrm{features}}+b_{4})+b_{5})+b_{6})$$

where the input $X_{\mathrm{level}2\_\mathrm{features}}$ denotes level_2 based features, $W_{4}$, $W_{5}$, and $W_{6}$ represent weights for three dense layers along with their corresponding bias terms $b_{4}$, $b_{5}$ and $b_{6}$. Moreover, σ(·) represents the sigmoid activation function used in the output layer to address multi-label classification.

## 5. Results

The hierarchical classification results for the two backbones are presented in this section. The classification performance for single-view (DV and LAT) is compared with multiview classification to demonstrate the efficacy of multiview image data. Interestingly, two architectures performed reasonably well for level_1 classification using single view, i.e., binary classification to differentiate *abnormal* specimens from *normal*. However, the advantage of multiview data becomes evident for the performance if granular classification at level_2 (three subclasses of abnormal) and level_3 (ten subclasses of abnormal), as demonstrated in this section. Moreover, it is worth noting that classification performance was evaluated using a threshold-free AUC metric, which defines a balance between true-positive rate and false0positive rate [26]. The area under the curve (AUC) quantifies the overall ability of the model to discriminate between positive and negative classes. An AUC value of 1 indicates perfect classification, while an AUC of 0.5 suggests performance no better than random chance. In practice, higher AUC values indicate better model performance. Since we are addressing the multi-label problem, selection of a particular threshold would not lead to realistic performance of the classification model [21]. Moreover, multi-label datasets often suffer from imbalanced distribution, i.e., some labels are less frequent. Hence, the standard accuracy metric can be misleading because a model truly predicting the majority class only will still reflect high accuracy.

### 5.1. ConvNeXt-Driven Hierarchical Model: Single vs. Multi-View

The ConvNeXt-driven hierarchical model was trained and validated level by level using cross-validation. For each level, the specimen-wise data was split into three subsets: training (70%), validation(20%), and testing (10%). Subsequently, the training and validation subsets were used to train models with early stopping criteria as explained in the implementation section. After completion of model training, the test subset (unseen images) was evaluated to assess performance at a respective level in terms of class-wise AUC score. Accordingly, level_1, level_2, and level_3 performance was computed using the respective test subsets. Moreover, the simulation was performed using five different seed values at each level, i.e., across different splits, to obtain the average performance for the respective level, as presented in Table 4.

Table 4 presents the performance of the hierarchical classification model for the single-versus-multiview representation of the specimen. The first column identifies the hierarchical level of abnormality, the second column precisely identifies the abnormality-subclass, whereas the subsequent three groups present the performance for the respective representation of the specimen (DV only, Lat only, multiview compact representation). The mean AUC values are presented as the five different splits were investigated, whereas the standard deviation represents consistency across five simulations. It can be observed that the model based on a single view led to a promising AUC score for the spine subclass (most prevalent); however, AUC scores for the remaining two subclasses were not very convincing (0.624 and 0.553 for DV) and (0.783 and 0.594 for Lat). In contrast, the last column, based upon multiview image data, shows improved performance for all three subclasses, validating the point that multiview image data leads to improved performance. Similar to level_2, we investigated the hierarchical performance of the model at level_3 for localized identification of structural abnormalities. The first two groups reveal that the model fails to perform very well for subclasses of abnormality using a single view. In contrast, the multi-view-based classification performance is presented in the last group and shows that the combined features from two views help the model to achieve improved classification capabilities.

**Figure 12:**
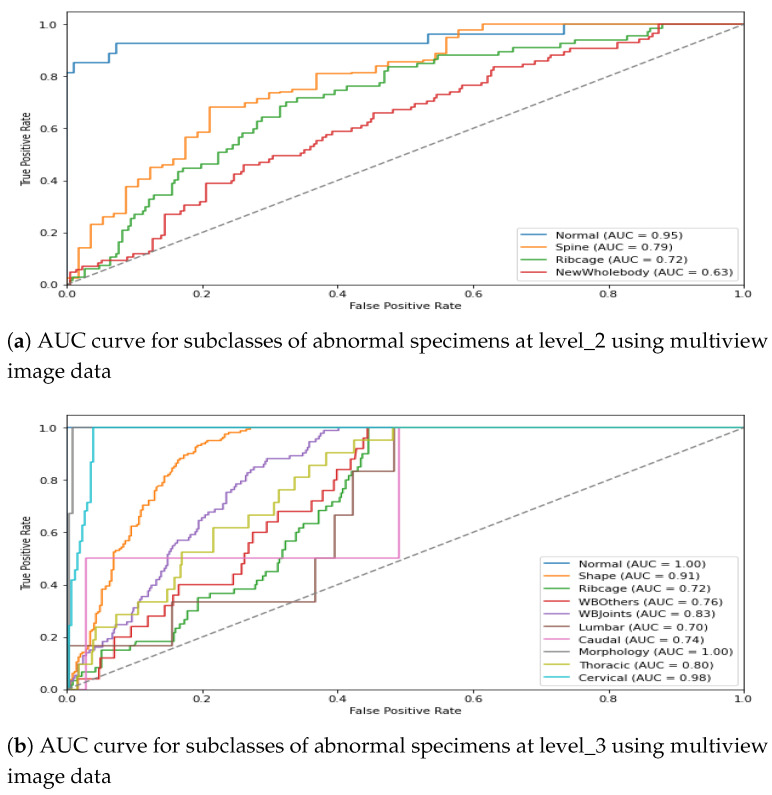
AUC score for multiview image data in the ConvNeXt-driven hierarchical model.

For the visual interpretation, the classification performance for two hierarchical levels using multiview representation is presented in Figure 12. It can be observed from Figure 12a that the model leads to a promising AuC score for three subclasses of *abnormal specimens* i.e., *Spine*, *Ribcage*, and *Whole-body*. Moreover, being the most prevalent class the in level_2 distribution, Spine shows a comparatively higher AuC score than do the remaining two subclasses. Similarly, Figure 12b shows the model performance for granular abnormalities at level_3; i.e., subclasses of *Spine* reflect a higher AuC score, which is as per expectations since spin-images were precisely classified in the preceding level. Similarly, the subclasses of Whole-body are also well detected at level_3, which remained very difficult in single view.

**Figure 13:**
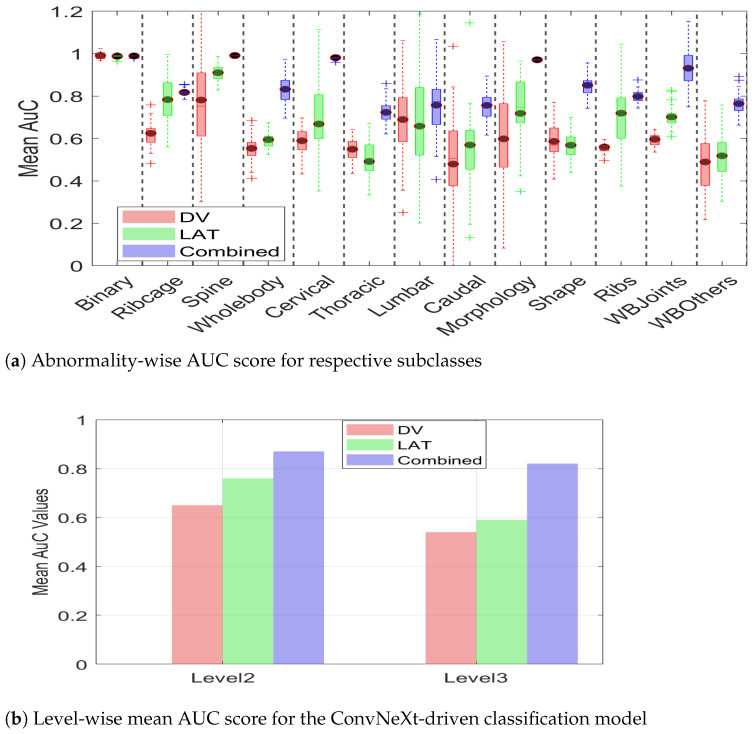
AUC scores demonstrating the advantage of multiview over DV and LAT. (**a**) Abnormality-wise AUC scores. (**b**) The mean AUC for the overall efficacy of multiview.

In addition to the quantitative summary presented in Table 4, we present a visual illustration of results using a Box plot in Figure 13 for brief comparison. It can be observed from the figure that model performance for binary (level_1) classification stayed almost the same for the DV, Lat, and combined views. However, the advantage associated with multiview data becomes apparent in level_2 and level_3 classification, where the combined view leads to overall improved performance in comparison with any of the single views.

Moreover, it can be observed from Figure 13a that multiview data not only leads to an improved AuC score but also leads to consistent performance, as evidenced by a reduced standard deviation. The smaller standard deviation further supports the notion that multiview data provides sufficient information, enabling the model to make more confident decisions. The summarized value indicating the cumulative mean for two hierarchical levels is shown in Figure 13b. It can be observed from the figure that the level_2 mean AuC for the DV, Lat, and multiview data is 0.65, 0.76, and 0.87, respectively. Similarly, the level_3 mean AUC for the DV, lat, and multiview representation is 0.54, 0.59, 0.82, respectively, which clearly demonstrates the efficacy of multiview data in hierarchical classification. The proposed hierarchical model was trained on a custom workstation with an AMD Ryzen-9 7900X 12-core processor with 32 GB of physical memory and an NVIDIA GeForce RTX 3090 Ti. With a potential training time of 280 min, the early stopping criteria often limit training time to 145–165 min. It is interesting to note that this model takes a comparatively longer training than does the CAE-driven architecture due to a high number of parameters.

### 5.2. XAI and Decision Interpretation

**Figure 14:**
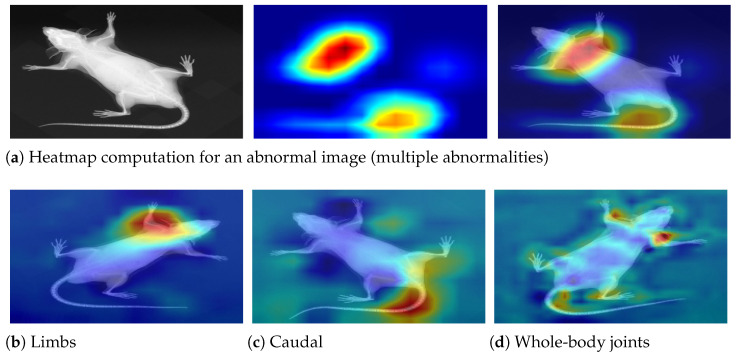
Decision interpretation using heat maps. (**a**) The step-wise process—input image, respective heat map, and superimposed image for a particular instance. (**b**–**d**) Three different specimens with limb, caudal, and joint abnormalities, respectively. It can be observed that the skeleton is precisely investigated in the relevant regions, leading to accurate predictions.

After performance evaluation of the classification model, we employed an explainability element to investigate the decision-making process. XAI (explainable AI) is often used for model optimization and debugging for enhanced performance by exploring regions of interest. Accordingly, we employed GRAD-CAM [29] visualization to highlight the image regions that most influenced the model’s predictions. The trained model was analyzed, and feature maps from the last convolutional layer were extracted. The weights associated with the predicted class were extracted from the final dense layer, and these weights were used to compute a class activation map by performing a weighted sum of the feature maps. The resulting activation map was then upsampled to match the original image size and normalized to a range between 0 and 1 for better visualization. Finally, the activation map was superimposed over the input image to visually highlight the regions that contributed most to the classification decision, as presented in Figure 14.

### 5.3. CAE-Driven Hierarchical Model: Single vs. Multiview

After demonstrating the advantage of multiview for ConvNeXt-driven hierarchical classification model, we present the performance of the CAE-driven hierarchical model. Similar to the ConvNeXt-based approach, the autoencoder hierarchical model was trained and subsequently used to classify the test subset level by level. The level_1 model was built using an autoencoder backbone with the motive of feature extraction using MSE optimization. Subsequently, the trained model led to a promising accuracy of 95% at the top level. For the level_2 classification, the dataset was split into training (70%), validation (20%), and test (10%) subsets. Accordingly, the level_2 model was trained to convergence using encoded feature maps, and the performance was evaluated over the test subset in terms of class-wise AUC score. Similarly, the level_3 model was built on top of the preceding level, and the performance was evaluated for the test subset. The experimentation was performed using five different seed values, i.e., across different splits, to obtain the average performance. The mean classification performance of the model at two hierarchical levels is presented in Table 5.

The first column presents the hierarchical level, the second column presents a specific subclass of abnormality, and the subsequent three groups present the classification performance (AuC score) for the DV, Lat, and multiview image data. It can be observed from the table that the binary classification to differentiate abnormal images from normal instances remained almost similar for the DV, Lat, and combined views. However, the advantage of multiview image data becomes evident for subclasses of level_2 and level_3. For instance, multiview image data leads to an improved AUC for the *Ribcage* and *Whole-body* subclasses, whereas *Spine* shows comparatively similar performance. Similar to level_2, we investigated the hierarchical performance of the model at level_3 for localized identification of structural abnormalities. The first two groups show that the model leads to reasonable accuracy for individual classes using a single view (DV or Lat); however, the mean AuC score increases substantially when multi- image data is used for classification. The increase in the mean AuC for granular abnormalities validates the assumption that multiview representation leads to overall improved classification performance of the hierarchical model.

**Figure 15:**
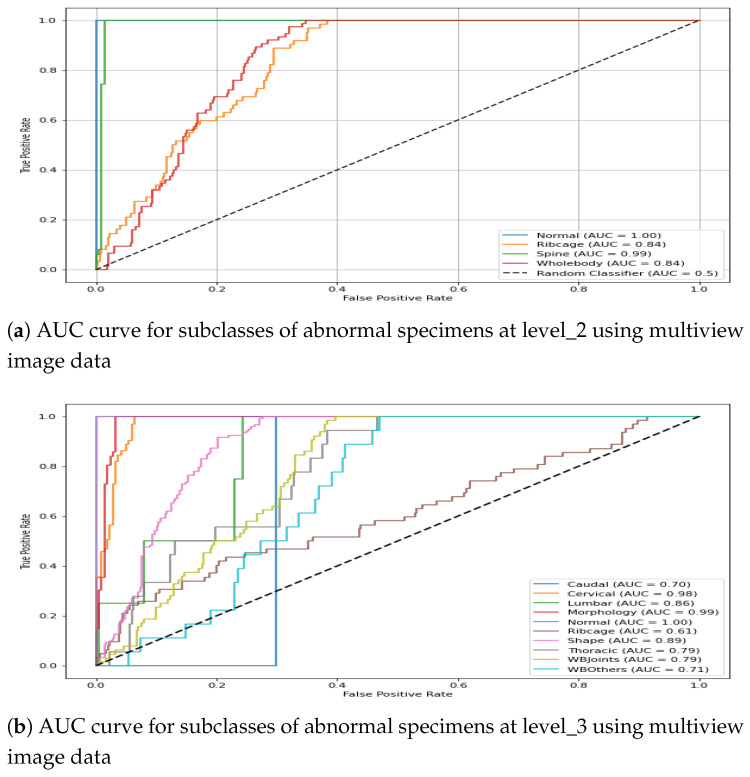
AUC score for multiview image data in the CAE-driven hierarchical model.

It is interesting to note that for some instances, the multiview-image-based AuC score drops in comparison with single-view performance. For instance, *Lumbar* subclass at level _3 shows mean AuC of 0.705 for DV, 0.756 for Lat and 0.723 for multi- image-based representation. This leads to the conclusion that if a single view already contains necessary distinguishing features for classification, the integration of additional views may not significantly improve performance; rather, multiview performance could drop due to potential redundancy of features or dominance of discriminative features in one particular view. The classification performance of the hierarchical model for the train–test split is presented graphically in Figure 15. The first plot shows the AuC curve for three subclasses at level_2, whereas the second plot represents the AuC curve for the granular 10 subclasses at level_3. It can be observed from the figure that the multiview-based level_2 model leads to an impressive AuC score for *Spine* and a comparatively good performance for *Ribcage Whole-body*. Similarly, the classification performance of the level_3 model shows a reasonable AuC for the majority of subclasses.

**Figure 16:**
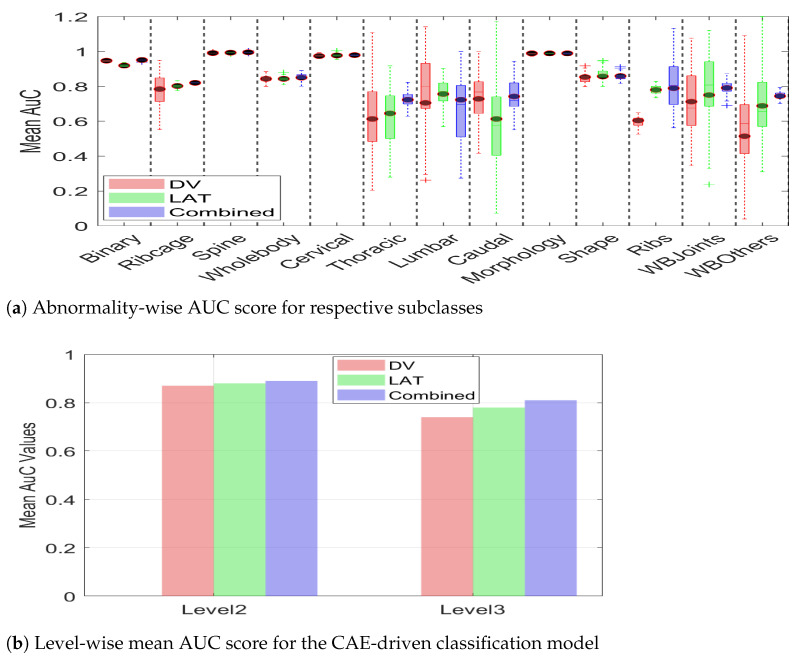
Mean AUC score for two classification levels with DV, LAT, and multiview image data. It can be observed from both (**a**,**b**) that multiview image data leads to improved classification in terms of a higher mean AUC score for both architectures.

The statistical analysis presented in the Table 5 is complemented with a box-plot representation of Figure 16. It can be observed from the figure that the AuC score for binary classification remains comparatively similar for the DV, LAT, and multiview images. However, the efficacy of multiview image integration becomes discernible in the detection of subclasses of abnormality at hierarchical levels. In addition to the improved mean AuC value for the majority of the subclasses, multiview image data also leads to a compact box, i.e., a decreased standard deviation. It can be observed that DV leads to substantial deviation for Thoracic, Lumbar, Caudal, WB-Others, whereas Lat leads to high variance for Thoracic, Caudal, and Shape. However, the multiview representation leads to minimized variance for all subclasses. This indicates that a combination of features from two different views leads to additional information, which helps the classification model to make consistent predictions. The summarized value indicating the cumulative mean for two hierarchical levels is shown in Figure 16b. It can be observed from the figure that the level_2 mean AuC for the DV, Lat, and multiview data is 0.87, 0.88, and 0.89, respectively. Similarly, the level_3 mean AUC for the DV, Lat, and multiview representation is 0.74, 0.78, and 0.81, respectively, which demonstrates the efficacy of multiview data in hierarchical classification. The proposed hierarchical model was trained on a custom workstation with an AMD Ryzen-9 7900X 12-core processor with 32 GB of physical memory and an NVIDIA GeForce RTX 3090 Ti. With a potential training time of 150 min, the early stopping criteria often limit training time to 65–75 min.

## 6. Discussion

This work demonstrated that multiview image data can lead to improved performance in comparison to any single view. The multiview performance was computed using two separate backbones, i.e., ConvNeXt and Convolutional Autoencoder architecture, with consistency in the classification results. Moreover, it is interesting to note that the convolutional autoencoder-based model performed comparatively better than did the ConvNeXT-driven model, which could be attributed to the efficient feature learning of the autoencoder architecture. It is also important to note that the dataset exhibits an uneven distribution of abnormalities across different views, which could lead to bias and overfitting and subsequently affect model generalization. However, we employed remedial strategies, including the selection of views and subsequent data augmentation strategies to minimize the impact.

An additional aspect for discussion is the inclusion of excluded views (hind legs, forepaws, and skull) for a more robust multiview representation of the specimen; however, these views are required for allspecimens, which is not the case in the IMPC data. As per the available IMPC image data, two full-body views are available for all specimens, whereas the additional views, including the skull, forepaws, and hind legs, are captured when required. Therefore, a six-view-based ideal multiview representation of mice specimens from the available dataset would lead to very few samples, which could negatively impact the robustness of hierarchical classification.

The future work aims to acquire missing views from the IMPC and subsequently extend the work by synchronizing six views for complete representation of every specimen; however, a substantial number of abnormalities in these additional views are needed. It is important to mention that the inclusion of additional views (skull, hind legs, and forepaws) can theoretically lead to better multiview representation; however, it may lead to increased computational complexity without any performance improvement if there are not sufficient structural abnormalities in these views.

## 7. Conclusions

In this work, we evaluated the potential of multiview representation of specimens for improved hierarchical classification. In general, abnormality detection is performed using a single-view radiographic image; however, additional abnormalities can be extracted if multiple views are integrated. Accordingly, we employed multiview X-ray data to represent individual specimens, followed by hierarchical classification. The hierarchical classification was performed using two customized architectures built on top of ConvNeXt and convolutional autoencoder backbones.The classification models were trained at different hierarchical levels, and their respective performance over the test subset was computed.

The results demonstrate the efficacy of the proposed multiview-based hierarchical model. At the top hierarchical level, the model achieved a comparable AUC score of approximately 0.95 for the DV, Lat, and multiview representations, indicating its strong ability to distinguish abnormal instances from normal ones. However, at the subsequent hierarchical levels, the advantages of multiview representation became evident. At level 2, the mean AUC values were 0.65, 0.76, and 0.87 for the DV, Lat, and multiview representations, respectively. Similarly, at level 3, the mean AUC scores were 0.54, 0.59, and 0.82 for the DV, Lat, and multiview representations, respectively, further highlighting the improved classification performance achieved by leveraging multiview data.

## Figures and Tables

**Table 1:** Different views and subsequent images for each viewpoint.

View	Number of Images	Normal	Abnormal	Total Abnormalities
Full body—DV	94,151	92,739	1412	3127
Full body—Lat	72,746	70,996	1750	5110
Skull—DV	36,709	36,695	14	29
Skull—Lat	29,830	9811	19	49
Forelimbs	26,257	26,249	8	45
Hindlimbs	1912	1903	9	46
Total	261,605	258,393	3212	8406

**Table 2:** Specimen-wise split of the collected dataset.

L1 Label	L2 Label	Number of Specimens
Abnormal	[Spine]	601
Abnormal	[Spine, Whole-body]	536
Abnormal	[Ribcage, Spine]	314
Abnormal	[Ribcage, Spine, Whole-body]	86
Abnormal	[Ribcage]	58
Abnormal	[Ribcage, Whole-body]	35
Abnormal	[Whole-body]	21
Total Abnormal count	-	1651

**Table 3:** Hyperparameters used in the two architectures.

Parameter	ConvNeXt-Driven	CAE-Driven
Optimizer	Adam	Adam
Loss	Binary cross-entropy	Binary cross-entropy
Epochs	100	100
Learning rate	0.00011	0.00001
Batch size	32	32
Early stopping criterion	Validation loss	Validation loss
Validation loss patience	3	3

**Table 4:** Mean performance for the ConvNeXt-driven hierarchical classification method.

	Abnormality	DV Only		Lat Only		Combined Multiview	
		Mean AuC	Std	Mean AuC	Std	Mean AuC	Std
Level_1	Binary	0.991	0.018	0.988	0.009	0.989	0.005
Level_ 2	Ribcage	0.624	0.046	0.783	0.122	0.817	0.017
	Spine	0.781	0.178	0.910	0.033	0.991	0.004
	Whole-body	0.553	0.065	0.594	0.032	0.832	0.069
Level_3	Cervical	0.589	0.066	0.668	0.144	0.981	0.008
	Thoracic	0.549	0.054	0.491	0.073	0.723	0.047
	Lumbar	0.689	0.167	0.658	0.249	0.757	0.159
	Caudal	0.479	0.211	0.569	0.173	0.756	0.071
	Morphology	0.598	0.176	0.718	0.149	0.971	0.005
	Shape	0.589	0.074	0.568	0.058	0.851	0.042
	Ribcage	0.559	0.020	0.719	0.141	0.798	0.028
	WB-Joints	0.596	0.028	0.701	0.048	0.831	0.075
	WB-Others	0.489	0.123	0.518	0.114	0.764	0.045

**Table 5:** Mean performance for the autoencoder-driven hierarchical classification method.

	Abnormality	DV Only		Lat Only		Combined Multi-View	
		Mean	Std	Mean	Std	Mean	Std
Level_1	Binary	0.947	0.006	0.919	0.008	0.951	0.005
Level_2	Ribcage	0.784	0.120	0.802	0.017	0.820	0.005
	Spine	0.991	0.004	0.993	0.003	0.995	0.003
	Whole-body	0.843	0.021	0.843	0.014	0.850	0.023
Level_3	Cervical	0.974	0.010	0.977	0.009	0.979	0.007
	Thoracic	0.613	0.247	0.645	0.142	0.723	0.041
	Lumbar	0.705	0.230	0.756	0.0801	0.723	0.190
	Caudal	0.728	0.143	0.613	0.267	0.742	0.102
	Morphology	0.989	0.006	0.989	0.005	0.989	0.006
	Shape	0.853	0.032	0.857	0.048	0.859	0.022
	Ribs	0.604	0.291	0.783	0.023	0.789	0.135
	WB-Joints	0.712	0.210	0.750	0.204	0.791	0.042
	WB-Others	0.514	0.241	0.688	0.192	0.744	0.021

## Data Availability

The image data used in this research is already available for public use at https://www.mousephenotype.org/, accessed on 28 April 2023.

## Abbreviations

The following abbreviations are used in this manuscript:

MSDs	musculoskeletal disorders
IMPC	International Mouse Phenotyping Consortium
DV	dorsoventral View
LV	lateral view
MV	multiview view
CNN	convolutional neural network
AUC	area under the curve
MSE	mean square error
CAE	convolutional autoencoder
